# 1950MHz Radio Frequency Electromagnetic Radiation Inhibits Testosterone Secretion of Mouse Leydig Cells

**DOI:** 10.3390/ijerph15010017

**Published:** 2017-12-23

**Authors:** Yan-Yun Lin, Tao Wu, Jun-Ye Liu, Peng Gao, Kang-Chu Li, Qi-Yan Guo, Meng Yuan, Hai-Yang Lang, Li-Hua Zeng, Guo-Zhen Guo

**Affiliations:** 1 Department of Radiation Medicine, Faculty of Preventive Medicine, The Fourth Military Medical University, Changle West Road 169, Xi’an 710032, China; fangyy@fmmu.edu.cn (Y.-Y.L.); summerlin0918@fmmu.edu.cn (T.W.); junyeliu@fmmu.edu.cn (J.-Y.L.); gaopeng1@fmmu.edu.cn (P.G.); fsyxjys1@fmmu.edu.cn (Q.-Y.G.); yuanboyue@outlook.com (M.Y.); Lsealhy@fmmu.edu.cn (H.-Y.L.); 2 Department of Radiation Biology, Faculty of Preventive Medicine, The Fourth Military Medical University, Changle West Road 169, Xi’an 710032, China; kangchu@fmmu.edu.cn

**Keywords:** 1950 MHz, radio frequency electromagnetic radiation, Leydig cell, testosterone

## Abstract

More studies that are focused on the bioeffects of radio-frequency (RF) electromagnetic radiation that is generated from the communication devices, but there were few reports with confirmed results about the bioeffects of RF radiation on reproductive cells. To explore the effects of 1950 MHz RF electromagnetic radiation (EMR) on mouse Leydig (TM3) cells. TM3 cells were irradiated or sham-irradiated continuously for 24 h by the specific absorption rate (SAR) 3 W/kg radiation. At 0, 1, 2, 3, 4, and 5 days after irradiation, cell proliferation was detected by cell counting kit-8 (CCK-8) method, cell cycle distribution, percentage of apoptosis, and cellular reactive oxygen species (ROS) were examined by flow cytometry, Testosterone level was measured using enzyme-linked immunosorbent assay (ELISA) assay, messenger ribonucleic acid (mRNA) expression level of steroidogenic acute regulatory protein (StAR) and P450scc in TM3 cells was detected by real-time polymerase chain reaction (PCR). After being irradiated for 24 h, cell proliferation obviously decreased and cell cycle distribution, secretion capacity of Testosterone, and P450scc mRNA level were reduced. While cell apoptosis, ROS, and StAR mRNA level did not change significantly. The current results indicated that 24 h of exposure at 1950 MHz 3 W/kg radiation could cause some adverse effects on TM3 cells proliferation and Testosterone secretion, further studies about the biological effects in the reproductive system that are induced by RF radiation are also needed.

## 1. Introduction

Radiofrequency (RF) electromagnetic radiation (EMR) is a non-ionizing radiation, with frequencies ranging from 10 MHz to 300 GHz that can have negative effects on human health [1,2]. Modern cellular phones typically operate at a frequency of 850 to 2100 MHz. While the biological effects of RF-EMR can be mediated through thermal and non-thermal mechanisms, it appears that the latter are more important in regards to cell phone usage [3]. In this context, the specific absorption rate (SAR) defines the amount of RF energy that is absorbed by body tissues and serves to evaluate the emission of transmitters located near the body [4]. In fact, the SAR resulting from cell phone RF radiation fluctuated between 0.12 and 1.6 W/kg of body weight [5], with the International Committee of Non-Ionizing Radiation Protection defining the threshold for safe exposure at 2 W/kg for both thermal and non-thermal effects, and at 4 W/kg for non-thermal effects [6].

A marked decline in male fertility has been reported in recent years [7,8]. Studies suggest that male infertility is mainly related to exposure to toxic chemicals, ionizing radiation, RF-radiation, and other environmental nuisances [9]. Moreover, several reports have linked cell phone usage with adverse effects on the male’s reproductive system [10]. Some of the negative reported effects of RF-EMR on testis function included increased permeability of the blood-testis barrier, changes in testicular morphology, decreased sperm count, motility, and percent normal morphology, as well as disrupted sperm DNA integrity or endocrine function [11]. Some of these studies suggested that the main mechanism that is linked to these unexpected effects was related to increased oxidative stress within the reproductive organs [12,13,14,15,16].

The studies of RF-EMR on testis function have mostly been performed in vivo level to evaluate gross effects on fertility or testis function, but have not given insight into the effects of this radiation in vitro level. In this regard, Leydig cells are the interstitial testis cells with a crucial role for supporting spermatogenesis and male reproductive function, given that they secrete 95% of the testosterone for the male [17]. Notably, in some studies, the application of RF-EMR radiation to mouse Leydig cells (TM3) resulted in upregulation of the Ets1 oncogene, providing a potential mechanism for the disruption of interstitial cell function [18]. However, no other parameters of cell function were evaluated in this study. Therefore, the objectives of our study were to investigate the effects of RF-EMR on the proliferation, viability, and function of TM3 Leydig cells.

## 2. Materials and Methods

### 2.1. Cell Cultureand Ethics Statement

TM3 cells that were used for our experiments were purchased from Shanghai Cell Bank of Chinese Academy of Sciences. Cells were expanded in gelatin-coated 10 cm Petri dishes. Medium consisted of Dulbecco’s modified Eagle’s medium (DMEM/ F-12 1:1; Invitrogen, Carlsbad, CA, USA), with 10% fetal bovine serum (FBS; Gibco, Grand Island, NY, USA). When cells reached about 80% confluence, they were harvested by 0.25% trypsin treatment (Invitrogen) and were used for experiments. Cell was always cultured in the incubator at 37 °C in a humidified air atmosphere with 5% CO_2_.

### 2.2. RF-EMR Exposure Equipment and Exposure Parameter

**Figure 1:**
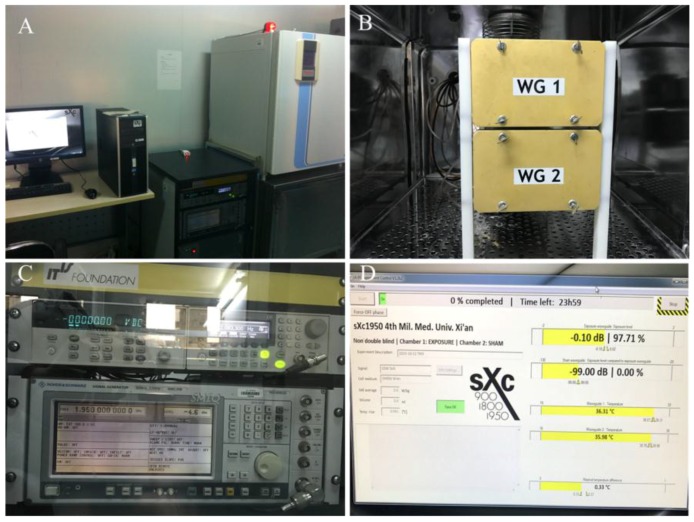
1950 MHz radiofrequency (RF) exposure system. (**A**) Exposure setup; (**B**) Mu-metal box; (**C**) Current source; and, (**D**) Arbitrary function generator controlled by a computer.

The exposure setups Xc-ELF irradiation system (sXc-1950, Zurich, Switzerland, Figure 1) that was used consists of four parts: the RF generator, the arbitrary function generator, the narrow band amplifier, and two rectangular waveguides (Zurich, Switzerland). Six standard 35-mm Petri dishes can be exposed in each rectangular waveguide, with one being used for irradiation and the second waveguide used for sham-exposure. The SAR (0~4 W/Kg) values are quantified with full feedback control in the exposure system. The sensors and fans of the exposure system are connected to a PC that monitors the SAR value (3 W/kg) during exposure and maintain a constant temperature and environment for the waveguides (37 °C, 5% CO_2_). The PC randomly selects one waveguide for exposure in each trial, and the temperature difference between the exposure and sham-exposure chambers does not exceed 0.1 °C. TM3 cells in the exponentially growing phase were used for experiments. For this purpose, 3 mL of cell suspension were seeded into 35 mm Petri dishes at the density of 1 × 10^4^ cells/mL, 4 h prior to placement into the exposure chambers. The dishes have been placed to fill both waveguides, and then, randomly, one set of dishes wastreated, and the other set was sham-exposed. The treated group received 1950 MHz GSM-Talk continuous signals for 24 h at SAR of 3 W/kg. Within the irradiation chambers, the temperature remained constant at 37 °C ± 0.091 °C during the exposure period.

### 2.3. Cell Proliferation Assay

After the 24 h treatment period, cells suspensions of exposed and sham-exposed groups were washed by centrifugation to remove trypsin, and then cells were resuspended in fresh DMEM medium. Cells suspension was then seeded into five pieces of 96-well dishes. In each dish, eight wells were seeded for each of the two groups (1000 cells/100 µL/well). Following culture for 24 h, 10 µL of the CCK-8 solution (Cell CountingKit-8, Bi Yun Tian, Nanjing, China) were added to each well and further incubated for 2 h, according to the manufacturer’s directions. The CCK-8 kit is based on the MTT assay, which relies on the transformation of MTT to form a non-water-soluble dye by the dehydrogenase activity within viable cells. Hence, the colorimetric quantification, which is performed at 450 nm using a spectrophotometer (Bio-Rad 680, Bio-Rad, Hercules, CA, USA), provides optical density values that correlate with the proportion of proliferating cells. Values averaging the optical density readings of five dishes for the exposure and sham-exposure group were used to make a proliferation curve.

### 2.4. Cell Cycle Analysis

As above, following the 24 h treatment period, the exposed and the sham-exposed cells suspensions were washed by centrifugation to remove trypsin and then cells have been resuspended in fresh DMEM medium. For each of the two groups, cells were distributed into 25 dishes, corresponding to five replicates at each of the following concentrations:1 × 10^5^, 0.5 × 10^5^, 1 × 10^4^, 0.5 × 10^4^, or 1 × 10^3^ cells/mL. Incubation proceeded for 1, 2, 3, 4, or 5 days, corresponding to each of the five concentrations seeded, respectively, to ensure that a similar number of cells would be available at each time period analysis. At harvest time, cells were washed twice with PBS and fixed in 70% ethanol at 4 °C, overnight. Then, cells were resuspended in PBS containing 0.01% RNase (Sigma-Aldrich, Saint Louis, MO, USA) and incubated with 0.5% propidium iodide at RT for 1 h. Cells were then analyzed by flow cytometry using a FACS Calibur Flow Cytometry at 488 nm (BD biosciences, Franklin Lakes, NJ, USA). The percentage of cells in different phases of the cell cycle was calculated by MultiCycle (DeNovo Software, Thornhill, ON, Canada).

### 2.5. Cell Apoptosis Analysis

Cells were processed as per cell cycle analysis up to harvest for analysis, which was also performed on day 1 to 5, following irradiation. The assay was performed using the Fluorescein Isothiocyanate Annexin V Apoptosis DetectionKit I (BD) following the manufacturer’s directions. Briefly, cells were harvested by trypsinization, washed twice with ice cold PBS, and then resuspended in 1× Binding Buffer at a concentration of 1 × 10^6^ cells/mL. Then, 100 µL of the cell suspension (1 × 10^5^ cells) were transferred to a 5 mL culture tube. Fluorescein Isothiocyanate Annexin V (5 µL) and propidium iodide (5 µL) were added, followed by gentle vortex and incubation for 15 min at RT in the dark. Then, 400 µL of 1× Binding Buffer were added to each tube without washing followed within 1 h by analysis using a FACS Calibur Flow Cytometer (Becton Dickinson, Franklin Lakes, NJ, USA). Gating was performed for FITC-Annexin V and/or PI staining, to identify the following potential groups: Negatively stained or viable cells; FITC-Annexin V^+^ but PI^−^ or early apoptotic viable cells; and, FITC-Annexin V^+^ and PI^+^ or apoptotic dead cells.

### 2.6. Testosterone Content Assay

Cells were processed to analyze Testosterone content after from day 1 to 5 consecutively irradiation. At those time points, the supernatant was collected, and cells were harvested by trypsinization, washed and placed in eppendorf tubes. After centrifugation, cell pellets were resuspended in 500 µL of lysis buffer (50 mM hydroxyethyl piperazine ethanesulfonic acid, pH 7.4; 5 mM propanesulfonate; 5 mM dithiothreitol) and incubated at 4 °C for 15 min. Extracts were then centrifuged at 14,000× *g* in a microfuge at 4 °C, and supernatants were transferred to fresh tubes. Protein concentration was quantified by the Bradford assay method using the Bio-Rad Dc System (Bio-Rad, Hercules, CA, USA). Then, Testosterone concentrations were determined by using an ELISA kit (Elabscience, Wuhan, China), following the manufacturer’s directions. Optical density (OD) measurements were performed as per cell cycle analysis.

### 2.7. StAR and P450scc mRNA Expression

Cells were processed to analyze StAR and P450scc mRNA expression after from day 1 to day 5 consecutively irradiation. At those time points, total RNA isolation was performed by using Trizol reagent, following the manufacturer’s directions. Then, 500 ng of total RNA were reverse-transcribed using the Real-Time Quantitative Reverse Transcription kit (Takara, Tokyo, Japan) in a 10 µL reaction volume following the manufacturer’s instructions. Then, 500 ng of DNA per reaction were used to detect different PCR products using specific primers to amplify the steroidogenic acute regulatory protein (StAR), cholesterol side-chain cleavage enzyme (P450scc), and glyceraldehyde 3-phosphate dehydrogenase (GAPDH). Cycling conditions consisted of 1 cycle of initial denaturation (95 °C, 30 s), and 40 cycles of amplification (95 °C, 5 s; 60 °C, 30~34 s). Primers were designed using the Primer Gene Synthesis software (Takara, Tokyo, Japan) with the following sequences: StAR (Forward) ACTCAACAACCAAGGAAGG, (Reverse) ATTTGGGTTCCACTCTCC; P450scc (Forward) AGAAGCTGGGCAACATGGAGTCAG, (Reverse) TCACATCCCAGGCAGCTGCATGGT; GAPDH (Forward) TCCTGCACCACCAACTGCTTAG, and (Reverse) AGTGGCAGTGATGGCATG GACT.

### 2.8. Intracellular Cell ROS Analysis

Cells were processed to analyze intracellular ROS after from day 1 to 5 consecutively irradiation. At those time points, cells from the exposure and sham-exposure group were handled as follows: 1 mL culture medium serum-free without serum was added into the each group after washing two times with PBS, (1) for the positive group, 1 µL Rosup was added into it and incubated at 37 °C for 20 min; (2) except the negative group, 1 mL diluted DCFH-DA (Reactive Oxygen Species Assay Kit, Jiancheng Bioengineering Institute, Nanjing, China) was added into each group, which based on 1:1000 scale, was diluted with serum-free culture medium, and then they were incubated for 20 min at 37 °C in the incubator; (3) During incubation process, these dishes were gently shaken in every interval of 3~5 min to make probe contact with the cells completely; (4) Then cells were washed three times in serum-free medium and intracellular ROS level was determined by flow cytometry.

### 2.9. Statistical Analysis

All data were expressed as the mean ± SD, and the analysis were carried by using SPSS 13.0 software (SPSS Inc., Chicago, IL, USA). All of the experiments were conducted at least in triplicate. Student *t*-test followed by homogeneity of variance test was used to analyze the significant differences between the exposed and sham-exposed group. The criterion for significance (*p*-value) was set at 0.05 and 0.01 compared the exposure with sham-exposure group.

## 3. Results

### 3.1. Proliferation of TM3 Cell was Inhibited by RF Radiation

**Figure 2:**
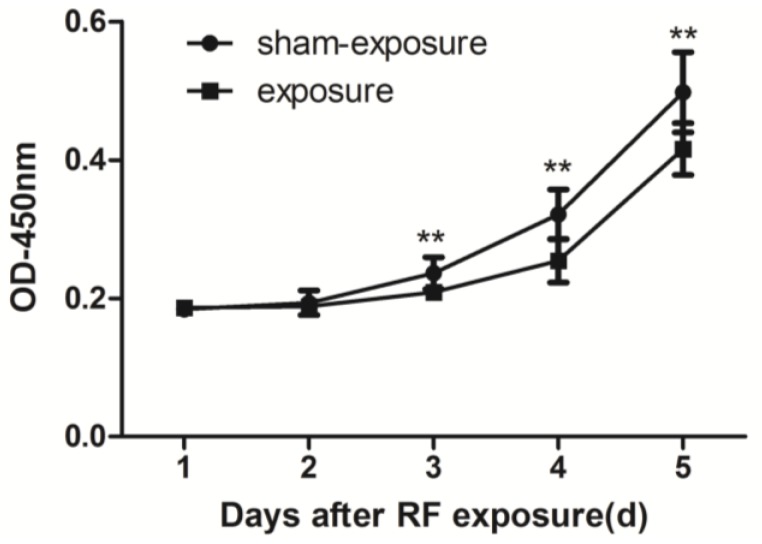
Proliferation of TM3 Leydig cells at day 1 to day 5 following exposure to radiofrequency (RF) electromagnetic radiation. Values represent mean ± SEM for each of the exposure and sham-exposure groups at each time point. ** *p* < 0.01 vs. sham-exposure (Student *t*-test).

By using CCK8 method assay, when compared to sham exposure group, cell proliferation in the exposure group was inhibited significantly at the day 3 to day 5 after 24 h of exposure at 3 W/kg 1950 MHz radiation (*p* < 0.01) (Figure 2).

### 3.2. RF Radiation Resulted in S Phase Arrest

When compared with the sham-exposed group, the proportion of G1 phase in TM3 cells decreased in the exposed group at 3 and 5 days after radiation (*p* < 0.05; Table 1). Conversely, the proportion of S phase increased in the exposure group at all time points after exposure (*p* < 0.05, Table 1). There were no significant differences in the proportion of cells in the G_2_ phase between the two groups (Table 1).

### 3.3. RF Radiation Produced No Effects on TM3 Cell Apoptosis

**Figure 3:**
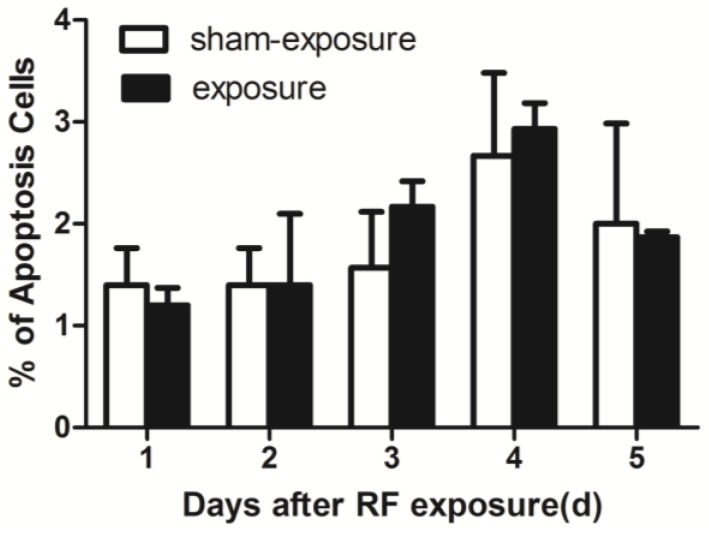
Percentage of apoptosis in TM3 Leydig cells at 1 day to 5 days following exposure to RF-EMR. Values represent mean ± SEM for each of the exposure and sham-exposure groups at each time point.

As compared to sham exposed group, percentage of cell apoptosis did not change at any of the post-exposure time points after present radiation by using Flow Cytometry assay (Figure 3).

### 3.4. Testosterone Secretion Inhibited by RF Radiation

**Figure 4:**
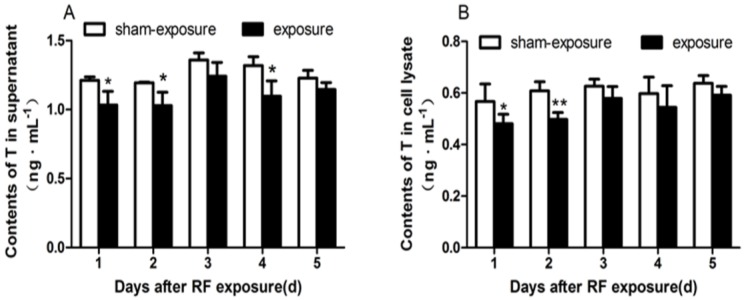
Testosterone contents in TM3 Leydig cells at day 1 to day 5 following exposure to RF-EMR. (**A**) Contents of T in the supernatant of TM3 cells; (**B**) Contents of T in the lysate of TM3 cells. Values represent mean ± SEM for each of the exposure and sham-exposure groups at each time point. ** *p* < 0.01 vs. sham-exposure (Student *t*-test), * *p* < 0.05 vs. sham-exposure (Student *t*-test).

Testosterone secretion is one of the most important function of Leydig cells. Testosterone contents were analyzed by ELISA assay. When compared to sham-exposed group, Testosterone contents in the supernatant were diminished at day 1, day 2, and day 4 following RF exposure (*p* < 0.05; Figure 4A). Similarly, as compared to sham-exposed group, intracellular Testosterone contents in the exposure group also decreased after the exposure, but there was significant difference only at day 1 and day 2 following the treatment (*p* < 0.05; Figure 4B).

### 3.5. StAR and P450scc mRNA Expression Downregulated by RF Radiation

**Figure 5:**
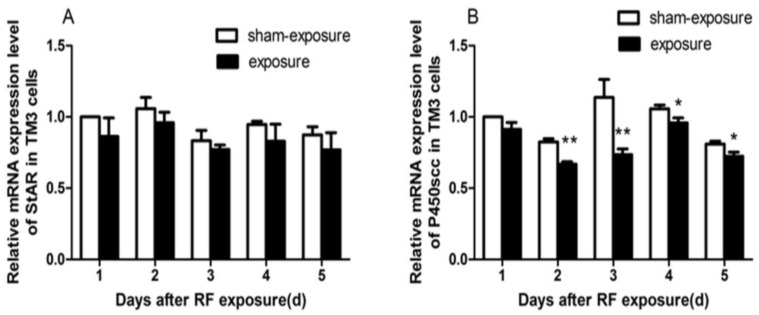
Effects of RF-EMR on mRNA expression of StAR and P450scc.(**A**) Relative mRNA expression levels of StAR induced by RF-EMR in TM3 cells; (**B**) Relative mRNA expression levels of P450scc induced by RF-EMR in TM3 cells. Values represent mean ± SEM for each of the exposure and sham-exposure groups at each time point. ** *p* < 0.01 vs. sham-exposure (Student *t*-test), * *p* < 0.05 vs. sham-exposure (Student *t*-test).

As compared to sham-exposed group, StAR and P450scc mRNA expression decreased in the exposure group (Figure 5), and only for P450scc mRNA expression, there were significant differences at day 2 to day 5 following the exposure (*p* < 0.05).

### 3.6. RF Radiation Produced No Effect on Intracellular ROS Generation

**Figure 6:**
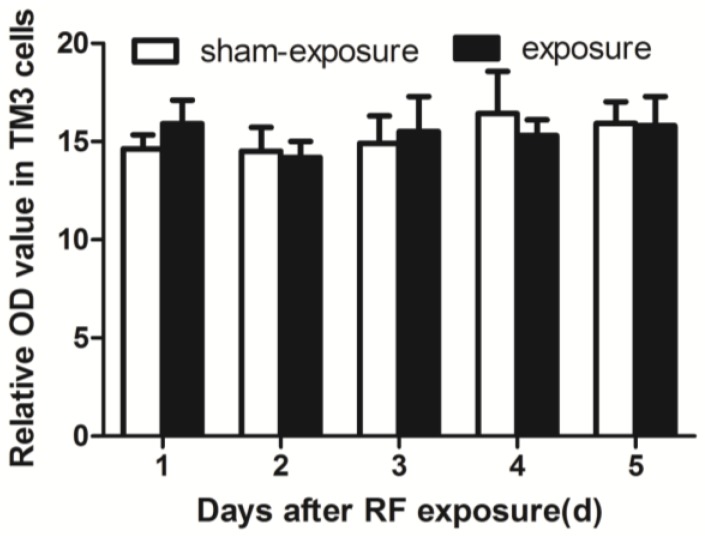
Effects of ROS levels in TM3 cells induced by RF-EMR. Values represent mean ± SEM for each of the exposure and sham-exposure groups at each time point.

By using flow cytometry assay, there were no significant differences in intracellular ROS levels between the exposure and the sham-exposed group (Figure 6).

## 4. Discussion

The interstitial cells of the testis, also termed Leydig cells, bestow an essential regulatory function upon male reproduction through testosterone secretion and consequent support spermatogenesis. Therefore, Leydig cells might be a target with negative effects of RF-EMR from cell phone usage on male reproduction from some researches [19,20,21,22,23]. Herein, we demonstrated that RF-EMR exposure at 1950 MHz negatively affected cell proliferation, cell cycle arrest, and testosterone secretion on TM3 cells, mouse Leydig cell line. It is consistent with previous in vivo studies, which changes of reproductive function induced by RF-EMR [19,21]. Other studies have also addressed the effects of RF-EMR of various frequencies, ranging from 50 MHz to 2.4 GHz different parameters of Leydig cells. For instance, Romano Spica et al. [18] reported an increase induction of the Ets1 oncogene in TM3 cells and postulated a negative effect on the cells’ activity related to Ca^2+^ metabolism, although this was not directly evaluated.

In the present study, when compared to the sham-exposed group, testosterone contents in the supernatant and intracellular of TM3, cells decreased significantly at different time points in the exposed group. Previous studies had shown swelling of intracellular organelles, such as mitochondria after Leydig cells that were exposed to RF-EMR [24,25], which might presumably affect their steroidogenic function. Notably, in the process of steroid synthesis, the conversion of cholesterol to pregnenolone is catalyzed by the enzyme cytochrome P450scc, which is localized on the matrix side of the inner mitochondrial membrane [26]. Then, the conversion of pregnenolone is catalyzed and transferred to testosterone by other steroid synthesis enzymes [27]. StAR is another important limiting enzyme in the synthesis of testosterone [28]. Consistent with the results which we found testosterone contents decreasing, the mRNA expression level of these enzymes such as P450scc and StAR was generally lower for exposed TM3 cells, although differences between the exposed and sham-exposed group only exists significantly from day 2 to day 5 for P450scc, following exposure.

In an attempt to understand the potential mechanisms behind the effects of RF-EMR on Leydig cells, we also assessed intracellular ROS generation. Several studies have hypothesized that the imbalance between intracellular free radicals and antioxidant systems leading to oxidative stress may play an important role in the process of damage stemming from different types of exposure [12,13,14,29,30,31]. Especially in some experiments in vivo, the results indicated that anti-oxidative stress could induce by electromagnetic radiation [32,33,34]. Also, there is some negative effects which RF-EMR couldn’t induce oxidative stress [35]. However, ROS levels in TM3 cells did not change significantly between the exposure group and the sham group after radiation, it suggested that oxidative injuries could not be induced by the present exposure condition.

Also, from our results, RF-EMR could not induce apoptosis in TM3 cells based upon the percentage of Annexin V^+^ or PI^+^ cells. In agreement, Zeni et al. [36] reported that 10 W/kg UMTS (Universal Mobile Telecommunications System) RF exposure for 24 h could not elicit any effects on cell viability and apoptosis in rat pheochromocytoma (PC12) cells. Conversely, in another study, the testes of male Wistar had a high percentage of apoptosis after exposure to 2.45 GHz microwaves and 900 MHz RF-EMF [20,37]. The power level of EMR (or SAR) is associated with the occurrence of DNA double strand breaks and the induction of apoptosis-related genes. Therefore, the discrepancies between this and our studies may be related to differences in RF radiation power and the sensitivity of different cell lines.

## 5. Conclusions

In summary, we found that it elicited inhibition of the proliferation, changes in cell cycle distribution, and the dysfunction of testosterone secretion in TM3 cells after exposure to 24 h continuous 1950 MHz GSM-Talk radiation with SAR of 3 W/kg. However, these effects were not induced by increasing of intracellular ROS levels and did not cause TM3 cells apoptosis. Hence, we hypothesize that inhibition of TM3 cell proliferation herein stemmed from changes in cell cycle distribution and lead to the dysfunction of testosterone secretion in TM3 cells in present studies. Further research is required to explore the molecular mechanisms involved in the suppression TM3 cell proliferation as well as testosterone secretion which induced by RF-EMR on male reproductive function.

## Figures and Tables

**Table 1:** Cell cycle distribution of TM3 cells after radiofrequency (RF) exposure.

Days	G1		S		G2	
	Sham	Exposure	Sham	Exposure	Sham	Exposure
1	45.56 ± 1.22	43.60 ± 1.54	26.75 ± 1.26	31.66 ± 1.00 **	27.69 ± 2.41	24.74 ± 2.19
2	51.16 ± 0.15	50.73 ± 2.41	20.18 ± 1.10	23.76 ± 0.47 **	28.66 ± 2.02	25.51 ± 2.46
3	51.44 ± 0.81	42.36 ± 3.77 *	27.49 ± 2.40	35.20 ± 2.28 *	21.07 ± 1.56	22.44 ± 1.1
4	49.46 ± 3.44	45.38 ± 1.44	25.62 ± 0.45	27.63 ± 1.06 *	24.92 ± 0.96	26.99 ± 1.04
5	55.38 ± 3.15	47.80 ± 0.84 *	20.14 ± 0.49	29.88 ± 2.53 **	24.21 ± 1.45	22.32 ± 0.83

Note: ** *p* < 0.01 vs. sham-exposure (Student *t*-test), * *p* < 0.05 vs. sham-exposure (Student *t*-test).
